# Supplementary material for: Collaborative intelligence in AI: Evaluating the performance of a council of AIs on the USMLE
Source: PLOS Digit Health. 2025 Oct 9;4(10):e0000787. doi: 10.1371/journal.pdig.0000787 (PMC12510544; doi:10.1371/journal.pdig.0000787)
Supplement: S2 File — (ZIP) [file pdig.0000787.s002.zip › S2 Data Raw data files containing unprocessed question inputs and LLM outputs including transcripts of deliberations/STEP3_Question-69-1_Type-MC-NJ.docx]

**Facilitator** **[Round 1]:** A 40-year-old woman comes to the office because of a 2-month history of fatigue, and generalized aching and weakness of the proximal muscles of all four extremities. The patient initially noticed the weakness only while she was getting in and out of her car, but during the past 2 weeks, the weakness has progressed, so that she now has difficulty combing her hair. Since the symptoms began, she also has had aching of the joints in her hands that has responded partially to ibuprofen. She was adopted and family history is unknown. She has two teenaged children who are well. She appears uncomfortable. She is 170 cm (5 ft 7 in) tall and weighs 68 kg (150 lb); BMI is 24 kg/m². Vital signs are temperature 37.7°C (99.8°F), pulse 90/min, respirations 20/min, and blood pressure 110/70 mm Hg. The patient is alert and fully oriented. Physical examination discloses cracking of the skin of both hands that involves the tips and lateral sides of several fingers. The muscles of the upper arms and legs are somewhat tender to pressure. Results of serum laboratory studies show a creatine kinase concentration of 600 U/L and a lactate dehydrogenase concentration of 800 U/L. Results of complete blood count are within the reference ranges. Which of the following is the most likely diagnosis? (A) Fibromyalgia (B) Myasthenia gravis (C) Polymyositis (D) Scleroderma

**AI_A:** RESTATING THE QUESTION: A 40-year-old woman has come to the clinic due to ongoing fatigue, generalized aching, and proximal muscle weakness. These symptoms, initially more subtle, now interfere with her ability to perform simple tasks such as combing her hair. She also reports hand joint pain, which improves somewhat with ibuprofen. There is no available family medical history as the patient was adopted. Her children are healthy. She's in obvious discomfort and presents with cracked skin on her hands, together with tenderness in her upper arm and leg muscles. Notably, her creatine kinase and lactate dehydrogenase levels are elevated, suggesting a myopathic condition. The question is about the most likely diagnosis: Fibromyalgia, Myasthenia gravis, Polymyositis, or Scleroderma. PLANNING: Several assumptions can be made based on these clinical findings. Firstly, the proximal muscle weakness, together with the elevated levels of muscle enzymes (creatine kinase and lactate dehydrogenase), suggest that the issue is likely muscular in nature, rather than neurologic. Secondly, the presence of joint pain and affected skin indicate an inflammatory process, possibly even a connective tissue disease. However, these issues might be separate entities. Evaluating each assumption, I would prioritize these symptoms' co-occurrence, supporting the hypothesis of a systemic disease rather than isolated conditions. CLINICAL REASONING: Muscle weakness, along with increased levels of muscle enzymes, usually points towards a condition causing muscle inflammation. Even though joint pain and skin involvement could be seen in different diseases, their presence in this case suggests an overlying connective tissue disorder. Fibromyalgia mainly presents with widespread pain, severe fatigue, and cognitive difficulties, without muscle enzymes elevation. Myasthenia gravis, a neuromuscular disorder, mainly causes weakness and rapid fatigue of any of the muscles under your voluntary control, commonly starting in the eye muscles. Polymyositis is indeed an inflammatory muscle disease causing muscle weakness, but it usually does not involve skin. CONCLUDING: Therefore, considering these aspects, the most plausible clinical diagnosis in this scenario is Scleroderma, a connective tissue disease that can involve the skin (manifested as skin cracking in this patient) as a form of sclerodactyly, the joints, and also the muscles causing aching and weakness. RESPONSE: The most likely diagnosis for this patient is (D) Scleroderma.

**AI_D:** RESTATING THE QUESTION: This patient, a 40-year-old woman, presents with generalized aching, fatigue, and progressed weakness specifically in the proximal muscles, muscle tenderness, and cracking skin of both hands over a period of two months. Her joint pain partially responded to ibuprofen. Her lab values suggest an elevated creatine kinase and lactate dehydrogenase, but the complete blood count is within normal limits. Family history is unknown due to adoption. The question is which of the four given diagnoses - Fibromyalgia, Myasthenia gravis, Polymyositis, Scleroderma - fits best with the described clinical picture. PLANNING: The key to unlocking this diagnosis lies in the examination of muscle weakness, skin changes, and the elevated levels of biochemical markers (creatinine kinase and lactate dehydrogenase). The common assumption is that these symptoms could be attributed to any muscle or connective tissue disorder. However, questioning this assumption may lead us to a more narrow alternative, specifically focusing on conditions that cause both muscle weakness and skin changes accompanied by elevations in creatinine kinase and lactate dehydrogenase. These markers point toward muscle tissue damage, which are more characteristic in certain muscle inflammation diseases. CLINICAL REASONING: Fibromyalgia typically presents with non-specific musculoskeletal pain and fatigue but does not cause skin changes or elevated muscle enzymes. Symptoms often respond to non-steroidal anti-inflammatory drugs (NSAIDs). Myasthenia gravis is an autoimmune disorder that affects the neuromuscular junction causing muscle weakness; nevertheless, it usually involves ocular muscles first and does not cause skin changes or elevate muscle enzymes. Scleroderma is a systemic sclerosis that could explain the skin changes and some muscle symptoms but would be unlikely to cause symmetric proximal muscle weakness and high muscle enzyme levels. Polymyositis, a type of idiopathic inflammatory myopathy, could explain all the features seen in this patient. It causes proximal muscle weakness, elevations in muscle enzymes, skin changes, and muscle tenderness. CONCLUDING: It is important to appropriately interpret the features of muscle weakness, skin changes, and elevated muscle enzyme values when making the diagnosis. The exact pattern of muscle weakness, the specific type of skin change, along with reactive elevation in muscle enzymes provide significant clues towards the most likely diagnosis. RESPONSE: The most probable diagnosis for the patient's condition, based on her clinical presentation and lab results, is (C) Polymyositis.

**AI_E:** Restating the question, a middle-aged woman presents at a healthcare facility with complaints of fatigue and generalized aching and weakness of the proximal muscles across all her limbs over the last two months. The symptoms initially surfaced when she was getting in and out of her car and have since escalated, now hindering her ability to comb her hair. Coinciding with these symptoms, she has also experienced aches in her hand joints that have modestly responded to ibuprofen. Her familial medical history is unknown due to being adopted. She's a mother of two teenagers who are in good health. Her body measurements and vital signs fall within the normal range. She presents with dried, cracked skin on both hands, primarily affecting the fingertips and lateral sides of several fingers, and tenderness in the muscles of the upper arms and legs. Laboratory tests indicate increased creatine kinase and lactate dehydrogenase concentrations. All parameters from complete blood count are normal. The objective is to identify the most likely diagnosis from fibromyalgia, myasthenia gravis, polymyositis, and scleroderma. Let's examine the options based on her symptoms and lab results. Fibromyalgia usually presents with widespread muscle pain and tenderness, but it's less likely in this case as it is not associated with increase in creatine kinase and lactate dehydrogenase. Myasthenia gravis is characterized by fluctuating muscle weakness, typically affecting ocular muscles first, and fatigue that worsens with activity, but unlike the patient's presentation, would not typically cause joint aches or rising enzyme levels. Polymyositis involves inflammation and weakness of skeletal muscles, particularly those closest to the trunk, and can cause elevated muscle enzyme levels, aligning with the patient's symptoms and lab results. Lastly, scleroderma involves tight, hard skin, potential muscle weakness, and may cause elevated muscle enzymes, however, the hand manifestations are usually manifested differently, with tightening and shiny skin. Clinically reasoning, the signs and pains indicate that the muscle inflammation could be the result of an autoimmune disorder, hinting a condition like polymyositis. The patient's joint ache and the hands' skin condition could be a sign of gravity-dependent edema that occurs in polymyositis. Considering the pattern of muscle weakness, it appears that proximal muscles are predominantly affected, which matches the symptom distribution in polymyositis. Fatigue and generalized ache are also common complaints in myositis-related disorders. Additionally, the lab results show elevation in muscle enzymes creatine kinase and lactate dehydrogenase, which are common in inflammatory myopathies due to muscle damage. From step-by-step reasoning, the patient's symptoms, physical examination, and laboratory findings align most accurately with polymyositis. The evidence supporting this diagnosis includes: proximal muscle weakness, generalized aching, increased muscle enzymes levels, and potential hands' skin changes. So, considering all the provided symptoms and laboratory results, the most likely diagnosis for this patient is Polymyositis (C).

**AI_C:** RESTATING THE QUESTION: To put this in other words, a 40-year-old woman is experiencing fatigue and muscle pain and weakness, particularly in the larger muscles close to her body's center, which has been gradually worsening over two months. She also experiences joint pain in her hands, partially relieved by ibuprofen, and her skin around her fingertips is cracking. Her blood work shows increased levels of both creatine kinase and lactate dehydrogenase. She has no known family history due to being adopted. We are attempting to determine the most appropriate diagnosis for this patient from the options of fibromyalgia, myasthenia gravis, polymyositis, or scleroderma. PLANNING: Firstly, we'll need to consider information about her symptoms, such as the muscle pain and weakness, fatigue, and skin changes. Secondly, the elevation of her creatine kinase and lactate dehydrogenase are diagnostic clues. A primary assumption might be that the patient's symptoms are related to a systemic disease rather than a localized issue alone. We should also consider if there is a secondary cause, like an autoimmune disorder, because of the patient's elevated muscle enzymes, which could imply musculoskeletal pathology. CLINICAL REASONING: While fibromyalgia, myasthenia gravis, and scleroderma could explain some of this patient's symptoms, these are less likely due to the absence of key classical manifestations or due to mechanisms unrelated to this patient's presentation. For instance, fibromyalgia is often seen with normal muscle enzyme levels; myasthenia gravis commonly presents with ocular symptoms and muscle weakness speared by repeated use; scleroderma, although it can cause skin changes, is usually linked with renal problems, pulmonary disease, or gastrointestinal symptoms, which this woman lacks. On the contrary, polymyositis is a systemic autoimmune disease characterized by inflammation and degeneration of the muscles, leading to muscle weakness and pain. Proximal muscles, those near the body's center, are usually the most affected. This symptom best fits her muscle weakness and joint pain around her hands, explained by an inflammatory process affecting her muscles and including her small joints. The elevation of creatine kinase and lactate dehydrogenase supports muscle damage, which aligns with the patient's complaint of muscle weakness and pain. CONCLUDING: Upon unraveling these clinical symptoms and lab findings, polymyositis fits this woman's picture. Her proximal muscle weakness, elevated muscle enzymes, pain relief with ibuprofen, and lack of other organs involvement suggest this diagnosis. RESPONSE: Therefore, the most likely diagnosis for this 40-year-old woman's presenting symptoms and lab results in the context of her medical history is (C) Polymyositis.

**AI_B:** RESTATING THE QUESTION: This patient, a 40-year-old woman, has been experiencing fatigue and muscle weakness in her extremities for the past two months. The symptoms first appeared during routine events like getting in and out of her car and have progressed to the point where she struggles with everyday tasks like combing her hair. She's also experiencing joint discomfort and her symptoms have been only partially alleviated by ibuprofen. Physical examination reveals skin cracking in her hands and tenderness in her upper arm and leg muscles. The creatine kinase and lactate dehydrogenase levels in her blood are higher than normal. Given these symptoms, we need to identify the most probable diagnosis from four options: (A) Fibromyalgia, (B) Myasthenia gravis, (C) Polymyositis, or (D) Scleroderma. PLANNING: The key assumptions here are the patient's symptoms, physical examination findings, and abnormal laboratory results. These assumptions could suggest various diagnoses, but we need to determine which diagnosis best fits the complete picture. For example, fibromyalgia is usually characterized by generalized pain and tenderness, but it doesn't explain the skin changes or elevated creatine kinase and lactate dehydrogenase levels. Myasthenia gravis would typically present with muscle weakness that worsens with repetitive use, and not necessarily with joint pain or skin changes. Polymyositis typically affects the proximal muscles and could explain the elevated creatine kinase levels, but again, it might not account for the skin findings. Lastly, scleroderma is a connective tissue disease that could explain the skin changes, but doesn’t typically manifest with such proximal muscle weakness or elevate creatine kinase levels. CLINICAL REASONING: As we evaluate each assumption, the clinical picture aligns most closely with polymyositis. Although it may be true that polymyositis does not typically present with skin changes, it's worth considering that this patient may manifest skin changes secondary to the inflammatory process inherent in the disease. The progression and distribution of her muscle weakness (affecting the proximal muscles), the pain and tenderness, and the elevation of muscle-related enzymes (creatine kinase and lactate dehydrogenase) are classic for polymyositis. This diagnosis also aligns with her relative non-responsiveness to ibuprofen, as NSAIDs are not generally effective in treating polymyositis, requiring more targeted immunomodulatory treatments. CONCLUDING: Thus, while some symptoms could be consistent with other pathologies, when taking into account the overall clinical picture along with the progression of the symptoms and the pattern of laboratory findings, polymyositis is the most likely diagnosis. RESPONSE: Thus, the most likely diagnosis for this 40-year-old woman experiencing a 2-month history of muscle weakness and aching is (C) Polymyositis.

**Facilitator** **[Round 2]:** Summary: AI_A believes the most likely diagnosis is Scleroderma (D), based on the patient's proximal muscle weakness, elevated muscle enzymes, joint pain, and skin changes. AI_A argues that these symptoms suggest a systemic disease, likely a connective tissue disorder, rather than isolated conditions. AI_B, AI_C, AI_D, and AI_E all believe the most likely diagnosis is Polymyositis (C). They all highlight the patient's proximal muscle weakness, elevated muscle enzymes, and joint pain as indicative of this condition. AI_B and AI_E also note that the patient's skin changes could be secondary to the inflammatory process inherent in Polymyositis. AI_C and AI_D argue that the other potential diagnoses do not fully account for all the patient's symptoms and lab results. Key Differences: The key difference in the council members' perspectives lies in their interpretation of the patient's skin changes. AI_A sees these changes as indicative of Scleroderma, a connective tissue disease that can involve the skin, joints, and muscles. In contrast, AI_B, AI_C, AI_D, and AI_E view the skin changes as potentially secondary to the inflammatory process in Polymyositis, a muscle inflammation disease. Question for Council Members: How do you interpret the patient's skin changes, and why do you believe they are more indicative of your chosen diagnosis (Scleroderma or Polymyositis) over the other?. Based on your preceding response, how would you answer the original question: [A 40-year-old woman comes to the office because of a 2-month history of fatigue, and generalized aching and weakness of the proximal muscles of all four extremities. The patient initially noticed the weakness only while she was getting in and out of her car, but during the past 2 weeks, the weakness has progressed, so that she now has difficulty combing her hair. Since the symptoms began, she also has had aching of the joints in her hands that has responded partially to ibuprofen. She was adopted and family history is unknown. She has two teenaged children who are well. She appears uncomfortable. She is 170 cm (5 ft 7 in) tall and weighs 68 kg (150 lb); BMI is 24 kg/m². Vital signs are temperature 37.7°C (99.8°F), pulse 90/min, respirations 20/min, and blood pressure 110/70 mm Hg. The patient is alert and fully oriented. Physical examination discloses cracking of the skin of both hands that involves the tips and lateral sides of several fingers. The muscles of the upper arms and legs are somewhat tender to pressure. Results of serum laboratory studies show a creatine kinase concentration of 600 U/L and a lactate dehydrogenase concentration of 800 U/L. Results of complete blood count are within the reference ranges. Which of the following is the most likely diagnosis? (A) Fibromyalgia (B) Myasthenia gravis (C) Polymyositis (D) Scleroderma]

**AI_D:** Restating the question: The complex scenario is about a 40-year-old woman who has come to a medical office due to her two-month experience of fatigue, generalized aching and weakness of proximal muscles and cracking of the skin of both hands. In her medical tests, she was found to have an elevated creatine kinase concentration and lactate dehydrogenase concentration. The task at hand is identifying her most likely diagnosis from the choices: Fibromyalgia (A), Myasthenia gravis (B), Polymyositis (C), and Scleroderma (D). Planning: There seems to be consensus among the council members on ruling out Fibromyalgia and Myasthenia gravis based on the presented symptoms. While Scleroderma and Polymyositis both could potentially align with the patient's symptoms, it is necessary to dissect the symptomatology and pathological processes of these conditions to identify what fits best. Clinical reasoning: The patient presents with proximal muscle weakness that has worsened over a two-month period, along with joint aching and skin cracking. This symptom profile strongly suggests an inflammatory muscle disease, like Polymyositis, which characteristically affects the proximal muscles and often is accompanied by elevated serum levels of muscle enzymes such as creatine kinase and lactate dehydrogenase. On the other hand, while Scleroderma can present with musculoskeletal symptoms, muscular involvement is less common and less likely to be the presenting feature. Moreover, skin changes involving the hands in Scleroderma typically features skin thickening and tightening, which is not the case with our patient who has skin cracking. Concluding: After evaluating the given patient symptoms, clinical signs, and lab results, Polymyositis seems to fit better than Scleroderma. Response: Considering the progressive proximal muscle weakness, generalized aching, increase in muscle-related enzymes, and response to ibuprofen indicating inflammatory process, the most likely diagnosis for the patient is Polymyositis (C).

**AI_B:** The question is asking which of the four given options—Fibromyalgia, Myasthenia gravis, Polymyositis, or Scleroderma—is most likely the diagnosis for this 40-year-old woman presenting with fatigue, muscle pain and weakness, joint aches, and specific changes to the skin of her hands, along with certain abnormal laboratory results. To answer this, we need to assess how well her symptoms align with the typical presentations of each of these conditions. Assumption number one could be that her symptoms sincerely represent Fibromyalgia or Myasthenia gravis, but those commonly do not display elevated muscle enzymes like creatine kinase and lactate dehydrogenase nor do they often present with skin changes. An alternate assumption is that the patient has a systemic illness affecting multiple organs and body systems, such as Polymyositis or Scleroderma. In evaluating these assumptions, it becomes clear that the latter possibility aligns better with the full range of the patient's symptoms and clinical findings. Taking a clinical reasoning approach, we can further analyze the two remaining conditions: Polymyositis and Scleroderma. Polymyositis, a type of inflammatory myopathy, is characterized by progressive muscle weakness, typically affecting the proximal muscles (as seen in the patient), muscular pain or tenderness, and often features raised muscle enzymes, which is seen in the patient's lab results. Scleroderma, a systemic sclerotic condition, also matches several of this patient's symptoms: it often presents with joint pain and skin changes like what we see here. However, the muscle involvement in Scleroderma is not usually as pronounced as what this patient is describing, and also Scleroderma commonly has other systemic features like GI symptoms, lung involvement, or Raynaud's phenomenon which are not present in this woman's case. Therefore, in conclusion, although Scleroderma might seem like a potential diagnosis due to joint pain and skin issues, the pronounced proximal muscle weakness in correlation with elevated muscle enzyme levels, matches more closely with Polymyositis. The skin changes experienced by the patient could be a manifestation of a related inflammatory process occurring within the body as a result of the Polymyositis. So, my final response to the scenario provided would be (C) Polymyositis.

**AI_C:** Firstly, understanding the patient's situation highlights her as a 40-year-old woman experiencing generalized fatigue, aching, muscle weakness – more pronounced in the proximal muscle groups of her limbs, and joint ache that partially responds to Ibuprofen. She has also reported that her symptoms have progressively worsened over a span of two months. Physical examination revealed skin changes including cracking of the skin on her hands. Laboratory findings disclosed elevated Creatine Kinase (CK) and lactate dehydrogenase (LDH) levels, enzymes commonly associated with muscle injury or inflammation. It's necessary to consider the progressive muscle weakness, joint pain, skin changes, and elevated muscle enzymes within our diagnostic contemplation. It's presumable that this woman is suffering from a systemic disease rather than localised conditions. Given the proximity of the muscle weakness and joint ache, it is plausible that our patient might suffer from a musculoskeletal condition. With elevated muscle enzymes coupled with muscle tenderness and proximal weakness on examination, conditions leading to muscle inflammation or injury become part of our differentials. But, the skin changes she has also raise suspicion for some form of connective tissue disease. Clinically, it would be essential to ascertain if the skin changes preceded or succeeded the muscle weakness and joint ache as this might alter our diagnostic trajectory. Evaluating the most likely diagnosis, Polymyositis (C) emerges as a potentially strong candidate. This condition is a form of inflammatory myopathy marked by muscle inflammation that predominantly affects the proximal muscles – a clinical feature that our patient exhibits. Moreover, Polymyositis often presents with elevated muscle enzymes (CK and LDH), which can correlate with the severity of the muscle inflammation. However, it's crucial to note that Polymyositis is typically not associated with skin symptoms, but a variant of the disease called Dermatomyositis is linked to skin changes. Numerous other conditions could account for some of the symptoms the patient is experiencing, albeit, not quite all. For instance, Fibromyalgia (A) could explain the fatigue, aching, and muscle weakness, but it would fail to account for the elevated muscle enzymes and skin changes. Myasthenia gravis (B) could cause muscle weakness, but its episodic nature and preponderant affectation of ocular and bulbar muscles makes it less likely. Scleroderma (D) could account for the skin changes and proximal muscle weakness; however, these patients typically present with 'Raynaud's Phenomenon' – a symptom not reported by our patient. Therefore, considering the general clinical picture, the most likely diagnosis is Polymyositis (C). Any adjoined skin changes could be due to Dermatomyositis. This conclusion is reached by examining the distinct symptomatology of the patient – the proximal muscle weakness, joint ache, skin changes, and raised CK and LDH – as well as methodically excluding alternative diagnoses which fail to cover the entirety of her symptoms. The optimal course of action would involve further diagnostic tests like muscle biopsy or MRI and consulting with a Rheumatologist.

**AI_A:** Restating the Question: The query at hand is aiming to determine a probable diagnosis for a 40-year-old woman who has been presenting for the past two months with fatigue, generalized aching, as well as proximal muscle weakness that has recently progressed. She also has joint pain in her hands and skin cracking on her fingers. All the noted symptoms have been examined in light of the absence of any known family history and her present vital signs. The list of potential diagnoses include Fibromyalgia, Myasthenia gravis, Polymyositis, and Scleroderma. Planning: All diagnoses presented overlap to a certain extent with the patient's symptoms. Each diagnosis is associated with pain and weakness, characteristics signified in the patient's situation, but diverge when it comes to the origin or nature of these symptoms. Thorough examination requires understanding that the primary difference between the potential diagnosis centers around connective tissue inflammation (as seen in Scleroderma), skeletal muscle inflammation (as seen in Polymyositis), a neuro-muscular junction disorder (as in Myasthenia gravis), or a non-inflammatory disorder characterized by musculoskeletal pain (as in Fibromyalgia). Additionally, the presence of aching joints and cracking skin further complicates the deductive process. Clinical Reasoning: The patient's proximal weakness is characteristic of a muscle disease rather than a neuromuscular or musculoskeletal disorder. This eliminates both Fibromyalgia and Myasthenia gravis from our priorities — the first is associated with pain more than weakness, and the second typically presents with ocular symptoms initially. Consequently, we are left to decide between Polymyositis and Scleroderma. Both can present with muscle weakness and pain but Scleroderma typically manifests skin symptoms, such as skin thickening and cracking, more commonly than Polymyositis. The muscle tenderness, joint aching, and increased muscle enzymes further support the idea of an inflammatory disorder. Concluding: The patient's symptoms involving several body systems including the skin, muscles, and joints, as well as her elevated muscle enzymes, lend more credence to a diagnosis of Scleroderma. This condition is a systemic autoimmune disorder that can lead to inflammation and fibrosis in the skin, vessels, and internal organs. In contrast, Polymyositis primarily involves muscle inflammation and would not explain the skin changes as evidently seen in the patient. Response: In conclusion, the most likely diagnosis for this patient is Scleroderma (D), a connective tissue disease that can cause a variety of symptoms including skin changes, joint pain, and muscle weakness. The patient's presentation is more consistent with Scleroderma rather than Polymyositis due to the presence of skin changes which are more distinctively associated with the former.

**AI_E:** The key clinical question here is to identify the most likely diagnosis for this 40-year-old woman, exhibiting symptoms of fatigue, generalized aching, and proximal muscle weakness, along with skin changes and elevated muscle enzymes. Her symptoms point towards pathologic muscular and dermal changes which could be a sign of a systemic disease. Firstly, the technical terms or concepts need to be clarified. Proximal muscle weakness refers to difficulties performing activities involving the large muscles close to the body's center, such as getting in and out of a car or combing one's hair. Elevated muscle enzymes, such as creatine kinase (CK) and lactate dehydrogenase (LDH), are indicative of muscle damage. The skin changes observed on this patient's hands, a symptom not commonly associated with muscle disorder, suggest a condition that affects multiple types of tissues. Considering these assumptions, let's evaluate the options given. Fibromyalgia (A) typically presents with widespread pain rather than localized to proximal muscles. While Myasthenia gravis (B) indeed causes muscle weakness, it does not cause elevation in muscle enzymes. Scleroderma (D) can cause skin changes and muscle weakness but it typically presents with a specific rash (not just cracking skin), Raynaud’s phenomenon (which this patient doesn’t appear to have), and systemic issues, like esophageal dysfunction or pulmonary fibrosis. Our alternate assumption is then that Polymyositis (C) might be the most clinically prioritized condition. Polymyositis can cause proximal muscle weakness, joint pain, elevated CK and LDH, as well as certain changes in the skin. Dermatomyositis, a related condition, could also manifest with skin and muscular symptoms, discoloration over the knuckles (Gottron's papules), or a heliotrope rash on the eyelids. Reviewing the laboratory findings, this patient presents elevated CK and LDH, suggesting muscle inflammation or injury, a characteristic of Polymyositis. The cracking observed in her skin may be due to dryness caused by systemic inflammation and while is not classic of a dermatomyositis rash, it does point to a systemic pathology. In conclusion, given the patient's symptoms, the progression of her condition, and the results of her lab tests, the most plausible diagnostic hypothesis is Polymyositis. It is a condition characterized by proximal muscle weakness and inflammatory muscle changes, aligning well with the presentation of our patient. Hence, the answer to the question will be (C) Polymyositis. Although it is necessary to consider other potential causes of the patient's symptoms, based on the given information, this diagnosis most comprehensively explains the clinical picture of proximal muscle weakness, skin changes and elevated muscle enzymes. Further testing, such as a muscle biopsy, can provide definitive diagnosis.

**Facilitator** **[Round 3]:** Summary: AI_A believes the most likely diagnosis for the patient is Scleroderma, due to the presence of skin changes, joint pain, and muscle weakness. The elevated muscle enzymes also support this diagnosis. AI_B, AI_C, AI_D, and AI_E, however, all conclude that the most likely diagnosis is Polymyositis. They argue that the patient's pronounced proximal muscle weakness, elevated muscle enzyme levels, and skin changes align more closely with Polymyositis. AI_C and AI_E also suggest that the skin changes could be a manifestation of Dermatomyositis, a variant of Polymyositis. Key Differences: The key difference in the council members' perspectives lies in their interpretation of the patient's skin changes. AI_A sees the skin changes as more indicative of Scleroderma, while AI_B, AI_C, AI_D, and AI_E view them as potentially related to Polymyositis or Dermatomyositis. AI_A also places more emphasis on the systemic nature of the patient's symptoms, while the others focus more on the muscle weakness and elevated muscle enzymes. Question for Council Members: How do you interpret the significance of the patient's skin changes in relation to the potential diagnoses of Scleroderma and Polymyositis? Could these changes be indicative of Dermatomyositis, a variant of Polymyositis, as suggested by AI_C and AI_E?. Based on your preceding response, how would you answer the original question: [A 40-year-old woman comes to the office because of a 2-month history of fatigue, and generalized aching and weakness of the proximal muscles of all four extremities. The patient initially noticed the weakness only while she was getting in and out of her car, but during the past 2 weeks, the weakness has progressed, so that she now has difficulty combing her hair. Since the symptoms began, she also has had aching of the joints in her hands that has responded partially to ibuprofen. She was adopted and family history is unknown. She has two teenaged children who are well. She appears uncomfortable. She is 170 cm (5 ft 7 in) tall and weighs 68 kg (150 lb); BMI is 24 kg/m². Vital signs are temperature 37.7°C (99.8°F), pulse 90/min, respirations 20/min, and blood pressure 110/70 mm Hg. The patient is alert and fully oriented. Physical examination discloses cracking of the skin of both hands that involves the tips and lateral sides of several fingers. The muscles of the upper arms and legs are somewhat tender to pressure. Results of serum laboratory studies show a creatine kinase concentration of 600 U/L and a lactate dehydrogenase concentration of 800 U/L. Results of complete blood count are within the reference ranges. Which of the following is the most likely diagnosis? (A) Fibromyalgia (B) Myasthenia gravis (C) Polymyositis (D) Scleroderma]

**AI_D:** RESTATING THE QUESTION: We have a 40-year-old female patient presenting with symptoms of weakness in the proximal muscles (those closest to the center of the body), fatigue, joint aches, and skin cracking. Her muscle weakness has progressively worsened to a point where it affects her daily activities such as combing her hair and getting out of a car. Additionally, her laboratory tests show elevated levels of creatine kinase and lactate dehydrogenase, enzymes usually elevated in muscle damage or inflammation. The question at hand is to ascertain the most likely diagnosis from among fibromyalgia, myasthenia gravis, polymyositis, and scleroderma. PLANNING: The assumptions made here are that the generalized symptoms could indicate systemic pathologies such as scleroderma or polymyositis. The skin symptoms seem to correlate with scleroderma, but can also link to polymyositis. The muscle weakness being more proximal may also suggest polymyositis or myasthenia gravis. Fibromyalgia often presents with fatigue and generalized pain, but the lack of a key symptom, tender points, along with the muscular enzyme changes might discount this option. CLINICAL REASONING: Scleroderma is a connective tissue disease that leads to hardening and tightening of the skin and connective tissues. The common symptoms are skin thickening, stiffness, and pain; however, the muscle weakness is less typical unless there is overlap with another connective tissue disease. Myasthenia Gravis, a neuromuscular disease, leads to varying degrees of skeletal muscle weakness; the most commonly affected muscles are those controlling eye and eyelid movement, which is not mentioned in this case. Fibromyalgia involves widespread musculoskeletal pain accompanied by fatigue, sleep, and mood issues, but wouldn't typically cause elevated creatine kinase and lactate dehydrogenase levels. CONCLUDING: Considering the broad systemic symptoms, the proximal muscle weakness, elevated muscle enzymes, aching joints, skin changes, and the progression pattern, polymyositis seems to be the most likely diagnosis. RESPONSE: (C) Polymyositis is the most likely diagnosis. Polymyositis is an inflammatory muscle disease causing symmetric muscle weakness and inflammation, explained by elevated muscle enzymes, and potential skin changes can be seen if overlapping with a variant like dermatomyositis.

**AI_B:** Restating the question: The fundamental question we are striving to answer here is: Based on the presenting symptoms, history, and laboratory findings of this 40-year-old woman, which of the listed disorders - Fibromyalgia, Myasthenia gravis, Polymyositis, or Scleroderma - is the most likely diagnosis? Planning: The symptoms presented — proximal muscle weakness, joint aching, skin changes, and elevated muscle enzymes — may be consistent with a range of disorders. The muscle weakness may indicate a neuromuscular disorder like Myasthenia gravis, but the absence of ocular symptoms and the skin changes make this unlikely. Fibromyalgia could explain the fatigue and muscle pain but would not account for the skin changes and elevated enzymes. Scleroderma may show skin changes but the muscle involvement is less typical. Polymyositis might explain the muscle weakness, skin changes, and elevated enzymes, but it would be crucial to consider if these skin changes could also indicate a variant of Polymyositis, like Dermatomyositis. Clinical Reasoning: While Myasthenia gravis and Fibromyalgia have elements that align with the patient's symptoms, they fall short in explaining the full range of presenting features. Similarly, Scleroderma, associated with skin thickening and systemic sclerosis, may explain some skin changes, but the pattern of widespread muscle weakness and elevated muscle enzymes are less characteristic. Polymyositis, characterized by proximal muscle weakness, elevated muscle enzymes (indicating muscle inflammation or damage), and potential skin changes, fits the patient's presentation more comprehensively. Though skin changes can occur in Polymyositis, they are more often associated with Dermatomyositis. However, without the characteristic heliotrope rash (blue-purple discoloration) around the eyes, Gottron's papules (red or violet bumps on the knuckles), or shawl sign (rash on the chest or back), it may be premature to classify this Polymyositis case as Dermatomyositis. Concluding: From a clinical standpoint, connecting these pieces of information, it appears that Polymyositis best fits the patient's presenting symptoms, physical examination findings, and lab tests. While skin changes in Dermatomyositis can't be ruled out at this stage, the absence of characteristic Dermatomyositis skin manifestations favors a diagnosis of Polymyositis over Dermatomyositis. Response: Therefore, based on the evidence at hand, I would answer choice (C) Polymyositis as the most likely diagnosis.

**AI_C:** RESTATING THE QUESTION: The question pertains to a 40-year-old woman who has been experiencing symptoms of fatigue, muscle weakness, and aching joints, along with changes to the skin of her hands, for about two months. While her blood count is normal, the concentrations of creatine kinase and lactate dehydrogenase enzymes in her serum are elevated. These two enzymes are typically associated with muscle damage. The question seeks the most likely diagnosis for her condition from among the options of Fibromyalgia, Myasthenia Gravis, Polymyositis, or Scleroderma. PLANNING: To answer this question, we must evaluate the symptoms, clinical signs, and laboratory findings presented. In particular, we should analyze the pattern and progression of the muscle weakness, the changes in the patient's skin, the partial relief of joint aches with ibuprofen, the normal blood count, and the elevated creatine kinase and lactate dehydrogenase levels. We need to determine which of these features are typical or atypical for each of the proposed diagnoses, and then identify the diagnosis that best fits the overall clinical picture. CLINICAL REASONING: The patient's symptoms suggest some form of systemic, likely autoimmune, condition. Both Fibromyalgia and Myasthenia Gravis are primarily characterized by muscle weakness, but the elevated enzyme levels in this case point to a more destructive process in the muscles, which is not typical of these two conditions. Additionally, the skin changes are not consistent with either of these diagnoses. The remaining plausible diagnoses are Polymyositis and Scleroderma. Scleroderma can cause skin changes, including cracking and hardening, but muscle weakness isn’t a usual predominant symptom and the muscle enzymes are not typically significantly raised. Polymyositis, on the other hand, is a systemic autoimmune condition that primarily affects the muscles, leading to proximal muscle weakness that worsens over time, just as described in this case. Importantly, it also typically causes elevations in muscle enzymes, reflecting ongoing muscle damage. The skin changes described for this patient are less typical for Polymyositis in its usual form, but they could potentially be a manifestation of Dermatomyositis, a variant of Polymyositis that also involves the skin. These variants form a spectrum, so it's not uncommon for a patient to present with features of both. CONCLUDING: Based on the constellation of symptoms described, the most likely diagnosis appears to be Polymyositis, potentially with features of Dermatomyositis given the skin changes. This diagnosis best explains the patient's clinical presentation, including the pattern of muscle weakness, the skin changes, and the elevated muscle enzyme levels. RESPONSE: Therefore, in answering the question: "Which of the following is the most likely diagnosis?" the answer should be; (C) Polymyositis.

**AI_E:** RESTATING THE QUESTION: This question presents a woman in her forties experiencing a series of symptoms including fatigue, muscle weakness, and muscle tenderness, that have progressively worsened over two months, along with joint aching. Moreover, unusual skin changes are apparent on her hands coupled with findings of elevated creatine kinase and lactate dehydrogenase levels. Given these clinical manifestations, the question requires identifying the most plausible diagnosis among the listed options: Fibromyalgia, Myasthenia gravis, Polymyositis, and Scleroderma. PLANNING: One assumption is that the skin changes are critical to the diagnosis. This could imply that the skin symptoms are linked to an underlying autoimmune condition given their co-occurrence with muscle weakness, fatigue, and elevated muscle enzyme levels. An alternative assumption is that these skin changes may be secondary manifestations of the primary disease process. When considering these assumptions, priority should be given to identifying associated patterns of disease that align with these clinical signs for a more accurate diagnosis. CLINICAL REASONING: The muscle weakness, especially noted during activities like climbing into a car or combing her hair, suggests a disorder impacting the proximal muscles. The elevated creatine kinase and lactate dehydrogenase levels concur with muscle damage or inflammation, as seen in muscular disorders. Fibromyalgia, which initially appears a valid assumption due to the fatigue and general muscle aching, is less likely given the elevated enzyme levels indicating active muscle damage, not typically seen in Fibromyalgia. Myasthenia gravis, characterized by fluctuating muscle weakness, often impacting ocular muscles first, doesn't fully align with the presentation either. The skin changes, consisting of cracked skin on the hands, often associated with autoimmune conditions, resume consideration of Polymyositis and Scleroderma. Scleroderma is classically associated with skin thickening rather than the cracking described. Polymyositis, on the other hand, aligns closely with the clinical picture: it is an inflammatory muscle disease associated with symmetrical proximal muscle weakness and elevated muscle enzymes. The skin changes could also suggest a variant of Polymyositis - Dermatomyositis, presenting with skin involvement alongside muscle inflammation. CONCLUDING: The patient's symptoms, notably the progressive proximal muscle weakness, elevated levels of muscle enzymes, and peculiar skin changes, can be interwoven into a coherent clinical picture consistent with the disease patterns of Polymyositis, including the possibility of its variant Dermatomyositis. RESPONSE: Therefore, after examining and evaluating the symptoms and clinical findings, the most likely diagnosis for this patient, given the provided options, is Polymyositis (C).

**AI_A:** To clarify, the patient is a 40-year-old woman presenting with generalized weakness, predominantly in the proximal muscles of all four limbs, progressive in nature, accompanied by aching of joints in her hands, skin changes on the hands and significantly elevated levels of muscle enzymes such as creatine kinase and lactate dehydrogenase. Based on the provided information, options for diagnosis include fibromyalgia, myasthenia gravis, polymyositis, and scleroderma. First, let's address the potentiality of each diagnosis. Fibromyalgia is characterized by widespread musculoskeletal pain accompanied with fatigue, sleep, memory and mood issues but it doesn't typically present with high levels of muscle enzymes or skin changes. Myasthenia gravis is initially characterized by Ocular Muscle weakness which could progress to generalized weakness. Fluctuating weakness of the voluntary skeletal muscles, especially those muscles that control eye movement and eyelid opening is the feature of myasthenia gravis. But our patient shows no sign of ocular weakness and has a high level of muscle enzymes, mainly seen in muscle inflammatory conditions. There might be the possibility of Polymyositis or Scleroderma. Polymyositis is a disease causing inflammation and progressive weakness of the muscles, especially those closest to the trunk. Symptoms include muscle weakness, fatigue, difficulty swallowing, and falls, and the condition is typically marked by elevated muscle enzymes. The skin changes, though not typical of polymyositis, could be a sign of Dermatomyositis which is a variant of the same disorder that involves a characteristic skin rash, and that seems objectively plausible. On the other hand, Scleroderma is a group of autoimmune diseases that may result in changes to the skin, blood vessels, muscles, and internal organs including muscle weakness, difficulty in breathing and swallowing, and skin changes like thickening, particularly of the hands and face. By linking the observable symptoms, examination findings, and lab results, the diagnosis can be directed towards Polymyositis. The reason behind this alternate assumption lies in the conjunction of significant proximal muscle weakness, elevated muscle enzymes which indicate muscle inflammation or damage and the nature of the progression of weakness within a relatively short period. The skin changes are atypical. By understanding the pathophysiology, we can then conclude that the elevated muscle enzymes, proximal muscle weakness, and the progressive nature of the disease tend to be more consistent with inflammatory muscle diseases, precisely Polymyositis or potentially Dermatomyositis. Therefore, my response is based on our previous discussion would be (C) Polymyositis as the primary diagnosis. The progressive nature of the disease, generalized proximal muscle weakness, and elevated muscle enzymes all point towards diagnosis B. Concurrent Dermatomyositis wouldn't be ruled out, considering the cracking of the skin of both hands, involving the tips and lateral sides of several fingers. However, more specific skin manifestations like Gottron's papules or heliotrope rash are usually expected in Dermatomyositis. It would be crucial to further investigate the skin manifestations for a more conclusive diagnosis. A muscle biopsy and further serological studies are also to be considered in such scenario to confirm the diagnosis. The possibility of Scleroderma should be pursued further if connective tissue disorder markers come positive in serology.

**Facilitator** The consensus among the AI responses is that the most likely diagnosis for the 40-year-old woman, given her symptoms and laboratory findings, is Polymyositis (C). The patient's symptoms of progressive proximal muscle weakness, elevated levels of muscle enzymes (creatine kinase and lactate dehydrogenase), and skin changes on her hands are consistent with this diagnosis. While Fibromyalgia and Myasthenia gravis were considered, they were ruled out due to the absence of key symptoms and the presence of elevated muscle enzymes, which are not typical for these conditions. Scleroderma was also considered due to the skin changes, but the muscle weakness and elevated muscle enzymes are less characteristic of this condition. Polymyositis, an inflammatory muscle disease, aligns closely with the patient's symptoms. It is characterized by symmetrical proximal muscle weakness and elevated muscle enzymes, indicating muscle inflammation or damage. The skin changes observed could suggest a variant of Polymyositis, Dermatomyositis, which presents with skin involvement alongside muscle inflammation. However, the absence of characteristic Dermatomyositis skin manifestations such as Gottron's papules or heliotrope rash makes this less likely. Further investigation, including a muscle biopsy and serological studies, would be beneficial to confirm the diagnosis. If connective tissue disorder markers are positive in serology, the possibility of Scleroderma should be pursued further.
